# Inhibition of Autophagy Suppresses Sertraline-Mediated Primary Ciliogenesis in Retinal Pigment Epithelium Cells

**DOI:** 10.1371/journal.pone.0118190

**Published:** 2015-02-11

**Authors:** Eun Sung Kim, Ji Hyun Shin, So Jung Park, Yoon Kyung Jo, Jae-Sung Kim, Il-Hwan Kang, Jung-Bum Nam, Doo-Young Chung, Yoonchul Cho, EunJoo H. Lee, Jong Wook Chang, Dong-Hyung Cho

**Affiliations:** 1 Graduate School of East-West Medical Science, Kyung Hee University, Yongin-si, Gyeonggi-do, Republic of Korea; 2 Division of Radiation Cancer Research, Korea Institute of Radiological and Medical Science, Seoul, Republic of Korea; 3 Research Institute for Future Medicine Stem Cell & Regenerative Medicine Center, Samsung Medical Center, Seoul, Republic of Korea; Sungkyunkwan University, KOREA, REPUBLIC OF

## Abstract

Primary cilia are conserved cellular organelles that regulate diverse signaling pathways. Autophagy is a complex process of cellular degradation and recycling of cytoplasmic proteins and organelles, and plays an important role in cellular homeostasis. Despite its potential importance, the role of autophagy in ciliogenesis is largely unknown. In this study, we identified sertraline as a regulator of autophagy and ciliogenesis. Sertraline, a known antidepressant, induced the growth of cilia and blocked the disassembly of cilia in htRPE cells. Following treatment of sertraline, there was an increase in the number of cells with autophagic puncta and LC3 protein conversion. In addition, both a decrease of ATG5 expression and the treatment of an autophagy inhibitor resulted in the suppression of the sertraline-induced activation of autophagy in htRPE cells. Interestingly, we found that genetic and chemical inhibition of autophagy attenuated the growth of primary cilia in htRPE cells. Taken together, our results suggest that the inhibition of autophagy suppresses sertraline-induced ciliogenesis.

## Introduction

Primary cilia are major cellular sensory organelles mediating the interaction between cells and external stimuli including chemical, mechanical, and paracrine signals. Structural and functional abnormalities of cilia are associated with various human diseases known as ciliopathies, such as Bardet-Biedl syndrome, neurosensory impairment, renal polycystic diseases, diabetes, hypertension, and cancer [1–3]. Therefore, the understanding of the regulation mechanism of ciliogenesis may useful in developing new therapeutic strategies against ciliopathies.

Cilia are maintained by intraflagellar transport (IFT) mechanism, which moves non-membrane-bound particles and building materials from the cell body to the growing cilium [4]. The IFT complex mediates anterograde and retrograde transport of proteins along the cilium. Primary cilia are implicated in the correct regulation of signal transductions including sonic hedgehog (SHH) and Wnt signaling [5,6]. The SHH transduction mechanism is regarded as a critical signaling pathway in the primary cilium. In this pathway, the SHH protein activates smoothened (Smo) by binding to its receptor, patched-1. The activation of the Smo signal transducer in turn activates Gli transcription factor, which induces the expression of genes such as those modulating renal patterning, cell cycle, and the Gli protein family. However, in the inhibition of primary cilia, the activation of Gli is suppressed, resulting in the turning off the SHH signaling [5]. In addition, both canonical and non-canonical Wnt signaling pathways have been reported to regulate cilium formation [7]. Further studies have reported that the cyclic AMP (cAMP) and NIMA-related kinase (NEK) family proteins regulates the assembly and disassembly of cilia. Activation of protein kinase A (PKA) by increased cAMP promotes assembly of the cilium, and congenital mutations on the NEK kinase proteins have resulted in ciliopathies [8, 9]. Moreover, the mammalian target of rapamycine (mTOR) signaling reversibly regulates ciliary length in zebrafish [10, 11]. The activation of mTOR induces cilia elongation while inhibition of the mTOR shortens the cilium length [12]. In addition, the status of the nutrient sensing mTOR pathway is closely linked to autophagy activation [13]. Furthermore, both autophagy and ciliogenesis are induced by serum deprivation, suggesting that autophagy may have a function in ciliogenesis [14].

Autophagy is a complex process of cellular degradation and recycling of cytoplasmic proteins and organelles, and plays an important role in cellular homeostasis. Therefore, the dysregulation of autophagy is highly associated with many pathological conditions including certain ciliopathies, such as neurodegenerative diseases and cancer [13, 15]. Despite its potential importance, the role of autophagy in ciliogenesis is largely unknown.

In this study, we screened the Prestwick chemical library and identified sertraline, an antidepressant of a selective serotonin reuptake inhibitor (SSRI) class as a potent inducer of autophagy and ciliogenesis. Sertraline treatment efficiently induced autophagy and ciliogenesis in human telomerase-immortalized retinal pigmented epithelial (htRPE) cells. In addition, inhibition of autophagy significantly suppressed the sertraline-mediated ciliogenesis in htRPE cells.

## Materials and Methods

### Reagents

Sertraline, 3-methyladenine (3MA), bafilomycin A1, and cytochalasin D were purchased from Sigma-Aldrich (St. Louis, MO). Ciliobrevin A1 was purchased from TOCRIS (St. Louis, MO). The expression plasmid pEGFP-Smo and pEGFP-LC3 (microtubule associated protein 1A/1B-light chain-3) were kindly provided by Dr. Kim, J (KAIST, Korea) and Dr. Noburu Mizushima (University of Tokyo, Japan). The previously validated small interfering RNA (siRNA) for human autophagy related gene 5 (ATG5) siRNA (5’- GCAACUCUGGAUGGGAUUG-3’) [16] and scrambled siRNA (5’-CCUACGCCACCAAUUUCGU-3’) were synthesized from Genolution (Seoul, Korea).

### Cell culture and stable cell line

Human telomerase-immortalized retinal pigmented epithelial (htRPE) cells and htRPE/Smo-GFP cells stably expressing Smo-GFP proteins were kindly provided by Dr. Kim, J (KAIST, Korea) [17,18]. The htRPE cells were maintained in Dulbecco’s modified Eagle’s medium (DMEM) supplemented with 10% fetal bovine serum, and 1% penicillin-streptomycin (Invitrogen, Carlsbad, CA). To generate a GFP-LC3 stable cell line (htRPE/GFP-LC3), htRPE cells were transfected with pGFP-LC3 and stable transfectants were selected by G418 (1 mg/ml) resistance following seven days treatment. After single cell dropping, stably expressing clones were selected under a fluorescence microscope. The m5-7 cells (ATG5 Tet-off system by doxycyline) were kindly provided by Noboru Mizushima (University of Tokyo, Japan) [19]

### Image-based chemical library screening

The htRPE/smo-GFP and htRPE/GFP-LC3 (3×10^3^) cells were seeded in 96-culture-well plate for the image-based chemical library screening. Following a 24 h incubation after seeding, each chemical of the Prestwick library (10 μM) (Prestwick Chemical, Illkirch, France) was added to each well. The cells were then cultured for a further 24 h, and cells with activated ciliogenesis and autophagy were observed under a fluorescence microscope (IX71, Olympus, Japan). Cytochalasin D was used as a positive control. The experiments were repeated twice with consistent results.

### Counting of autophagic cells and confocal microscopy

The cells were treated with sertraline (10 μM), and the number of autophagic cells were determined by counting the number of cells with GFP-LC3 punctuate structures under a fluorescence microscope. For confocal microscopy, htRPE/smo-GFP or htRPE cells were plated on glass-bottom dishes. Cells were pre-treated with 3MA (5 mM) and ciliobrevin A1 (10 μM). After 12 h, the cells were treated with Sertraline (10 μM) and cytochalasin D (50 nM). After 24 h, the cilia were visualized with Smo-GFP or immune-stained with poly glutamylated tubulin antibody (Adipogen, San Diego, CA), and the images were captured using a confocal laser scanning microscope (LSM510) (Carl Zeiss Microimaging Inc., Thornwood, NY).

### Measurement of increased cilium number and cilium length

The htRPE/smo-GFP cells transfected with siRNA were treated with sertraline (10 μM) for 24 h. Changes in the cilium numbers were measured by counting the cilia under a fluorescence microscope (IX71, Olympus, Japan). The cilium length was measured using the ‘cellSense’ Standard software (Olympus, Hamburg, Germany). The average cilium length was determined using the Free-hand Line Selection Tool. The length of an individual cilium was obtained from randomly selected cells. And the images were analyzed and digitized using the cellSense Standard software (≥15 cells per experiments, n = 3).

### Western blot analysis

Whole cell lysates were prepared with protein sample buffer (62.5 mM Tris-HCl, pH 6.8, 25% glycerol, 2% SDS, 5% β-mercaptoethanol, 0.01% Bromophenol blue) (BioRad, Hercules, CA). After separation in 10–15% SDS-PAGE, proteins were transferred onto polyvinylidene fluoride membrane and (Bio-Rad, Hercules, CA). The membranes were blocked with 4% skim milk in TBST for 1hr and then incubated with specific primary antibodies overnight at 4°C. Anti-ATG5 (ab54033, 1:2000) antibody was purchased from Abcam (Cambridge, UK); anti-LC3 (NB100-2220, 1:10,000) antibody was purchased from NOVUS Biologicals (Littleton, CO); p62 (Sc-28359) antibody was obtained from Santa Cruz Biotechnology (Santa Cruz, CA); anti-Actin (MAB1501, 1:10,000) antibody was purchased from Millipore (Temecula, CA). For protein detection, the membranes were incubated with HRP-conjugated secondary antibodies and signals were detected with Super-signal West Dura HRP detection kit (Pierce, Rockford, IL).

### Statistical analysis

Data were obtained from least three independent experiments, and presented as means ± S.E.M. Statistical evaluation of the results was performed with one-way ANOVA (*p<0.05).

## Results

### Sertraline induces the formation of primary cilia by suppressing cilia disassembly in htRPE cells

**Fig 1:**
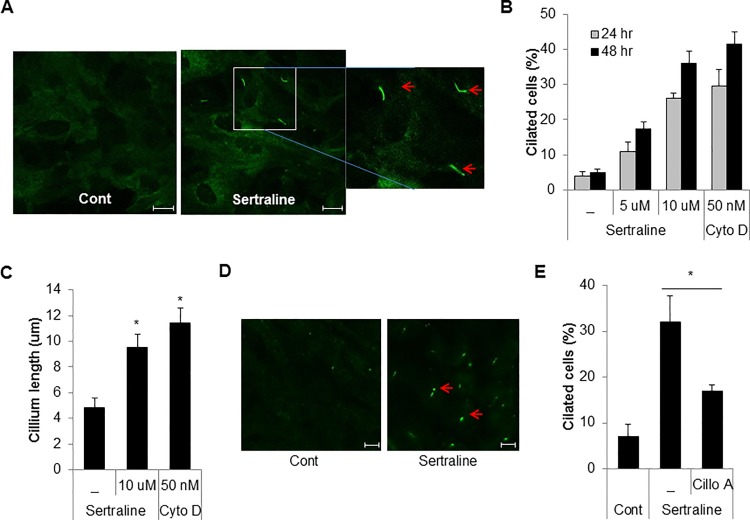
Sertraline induces primary cilia formation in htRPE cells. (A) htRPE/Smo-GFP cells were treated with or without sertraline (10 μM) for 24 hr. Then, the cells were imaged by confocal microscopy. Arrows indicate the primary cilia (Scale bars: 10 μm). (B and C) htRPE/Smo-GFP cells were treated with sertraline (10 μM) or cytochalasin D (50 nM). The increased ciliated cells and the cilium length of the cells were measured. (D) htRPE cells treated with sertraline (10 μM) were stained with anti-polyglutamylated tubulin antibody. Arrows indicate the primary cilia (Scale bars: 10 μm). (E) htRPE/Smo-GFP cells pre-treated with ciliobrevine A1 (Cilio A) (10 μM) for 1 h were further exposed to sertraline (10 μM) for 24 h. Then the ciliated cells were counted under a fluorescence microscope. Data were obtained from at least three independent experiments and values are presented as the means ± S.E.M. (n = 3, * *p* < 0.02)

**Fig 2:**
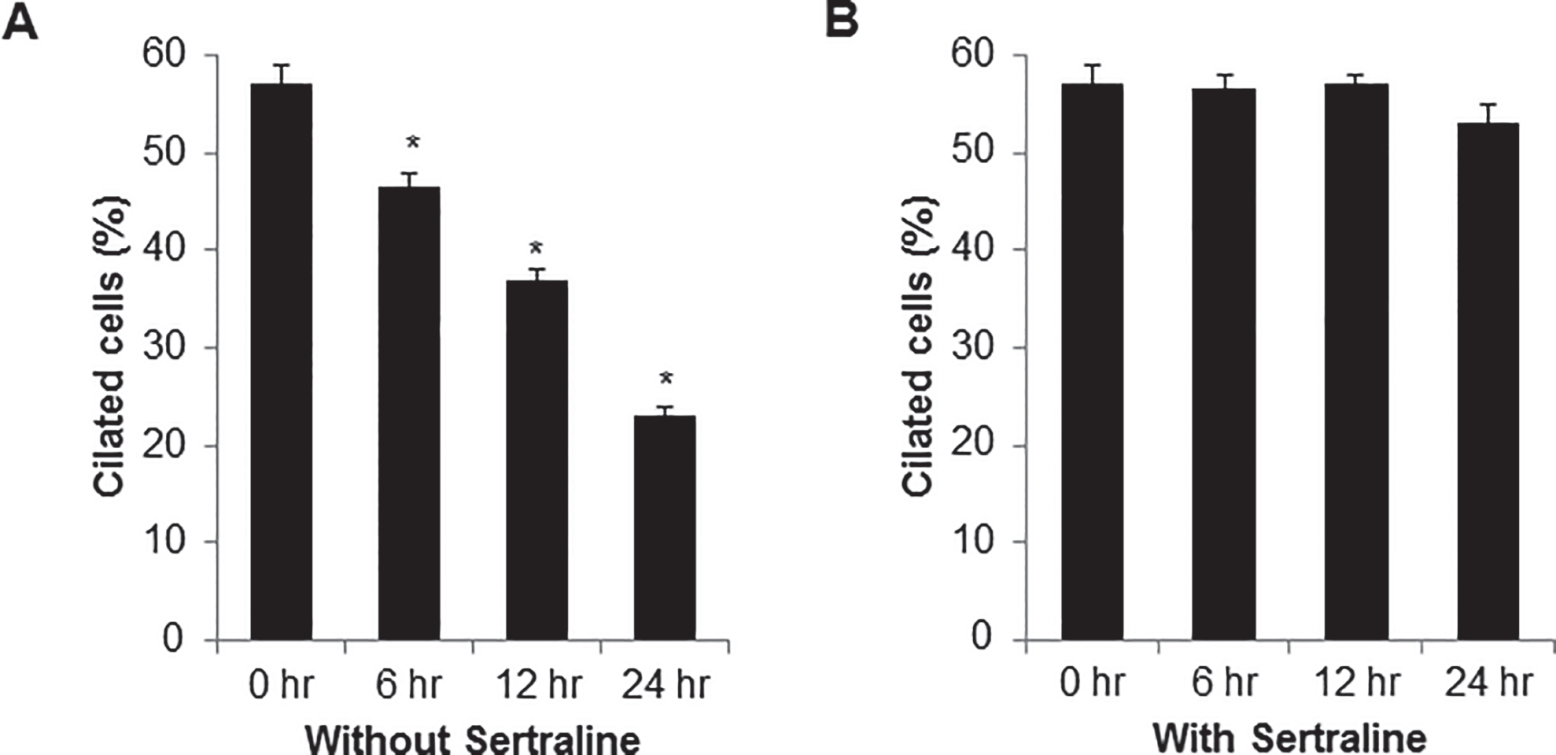
Sertraline inhibits primary cilia disassembly in htRPE cells. (A and B) htRPE/Smo-GFP cells were plated in a medium without serum for 48 h. Next, the cells were further incubated with normal medium in the absence (A) or presence of sertraline (10 μM) (B). The percentage of cells with cilia was counted at the indicated time points. Data represent ± standard error of the mean (S.E.M.) from three independent experiments (n = 3,* *p* < 0.02).

Cilia are complex polarized structures that associated with several cellular processes such as the cell cycle, migration, polarization, development of vertebrate and genetic diseases [7,20]. The Smo protein is accumulated on the primary cilium and is widely used as a cilium marker [21]. To identify the chemical modulator of ciliogenesis, we developed a cell-based screening system using htRPE cells that stably expressing a fluorescent protein fused with Smo (htRPE/Smo-GFP) [17,18]. Using this screening system, we screened the Prestwick chemical compound library, which is composed of market drugs. As a result of the screening process, we selected sertraline for further analysis as a potent regulator of ciliogenesis. Sertraline is SSRI anti-depressant with a high binding affinity towards the serotonin transporter. Sertraline is predominantly prescribed for depression and obsessive-compulsive disorder [22]. However to date, the effect of sertraline on ciliogenesis has not been fully addressed. To confirm the screening results, htRPE/Smo-GFP cells were treated with sertraline and the cells were observed by fluorescence microscopy. As shown in Fig. 1, treatment of sertraline strongly increased the formation of primary cilia (Fig. 1A and 1B). Previously it has been shown that cytochalasin D (Cyto D) highly induced cilium formation by inhibiting actin polymerization whereas, ciliobrevin A1 (Cilio A1) inhibited ciliogenesis by suppressing cytoplasmic dynein [17, 23]. From our data, treatment with sertraline greatly elongated the cilium length as much as Cyto D in htRPE cells (Fig. 1C). Several post-translational modifications (PTM) are involved in regulation of the ciliary tubulin, and the poly-glutamylation of tubulin is the predominant PTM [24]. According to the previous result, treatment with sertraline in htRPE/Smo-GFP cells also highly induced the levels of poly-glutamylated tubulin (Fig. 1D). Moreover, treatment of Cilio A1 significantly reduced the sertraline-mediated ciliogenesis (Fig. 1E), indicating that sertraline induces primary cilium formation in htRPE cells. In theory, cilium elongation could be induced by both increased assembly and decreased disassembly of cilia. Therefore, we explored further the effect of sertraline on cilia assembly and disassembly. Cilium elongation was induced by culturing the htRPE/Smo-GFP cells without serum for 48 h, since serum starvation promotes elongation of the cilia [25]. Then, the cells were incubated in a normal medium in the presence or absence of sertraline. Re-feeding the cells with serum gradually disassembled the cilia over a period of 24 h. (Fig. 2A). However, the disassembly of the cilia was almost completely prevented in the sertraline-treated cells (Fig. 2B), suggesting that sertraline increase ciliated cells by suppressing the disassembly of the primary cilium in htRPE cells.

### Sertraline induces autophagy in htRPE cells

**Fig 3:**
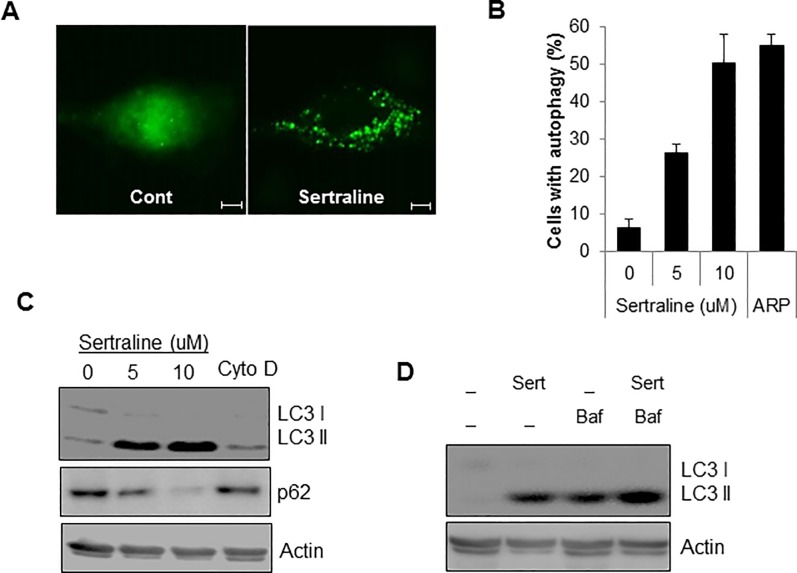
Sertraline activates autophagy in htRPE cells. (A) htRPE/GFP-LC3 cells were treated with sertraline (10 uM) and fixed to for the fluorescence imaging (Scale bars: 20 μm). (B and C) htRPE/GFP-LC3 cells were exposed to either increasing concentration of sertraline (5, 10 μM) or cytochalasin D (Cyto D, 50 nM) for 24 h. Cells with autophagic punctuate structures were counted (B). Cells were harvested to analyze Western blotting with indicated antibodies (C). (D) htRPE cells were treated with Sertraline (Ser, 10 μM) with or without an autophagy inhibitor, bafilomycin A1 (Baf). The expression level of LC3 protein was detected by Western blotting. Data were obtained from at least three independent experiments and values are presented as the means ± S.E.M. (n > 3, * *p* < 0.02).

Serum starvation induces both autophagy and the formation of primary cilia. Therefore, we also addressed the effect of sertraline on autophagy in retinal pigment cells. The LC3 protein is wildly used as a molecular marker to detect autophagosome formation. During autophagy activation, LC3| is converted to LC3|| [13]. Thus, we verified autophagy activation by observing the change of LC3 protein. Treatment of sertraline increased the presence of autophagic GFP-LC3 punctate structures in a dose-dependent manner in htRPE cells (Fig. 3A and 3B), where ARP101 was used as a positive control for autophagy activation [26]. Additionally, the LC3|| protein was highly increased in sertraline-treated cells but not in Cyto D-treated cells (Fig. 3C). Furthermore, we investigated the effect of sertraline on autophagic flux, since the inhibition of autophagic flux in the fusion process of autophagosomes to lysosome, as well as autophagy activation, could induce the accumation of autophagosomes. Accordingly, we examined the expression of p62 protein, in sertraline-treated cells, since p62 is incorporated into autophagosome and selectively degraded in lysosome [13]. As shown in Fig. 3C, p62 is down-regulated following sertraline treatment, suggesting that sertraline induces autophagic flux. In addition, co-treating cells with a lysosome inhibitor, bafilomycin A1, confirmed the activation of autophagic flux by sertraline. Combination treatment with sertraline and bafilomycin A1 resulted higher levels of LC3|| than the treatment with sertraline alone did (Fig. 3D). Taken together, these results suggested that sertraline induces the activation of autophagy in htRPE cells.

### ATG5 regulates sertraline-mediated autophagy in htRPE cells

**Fig 4:**
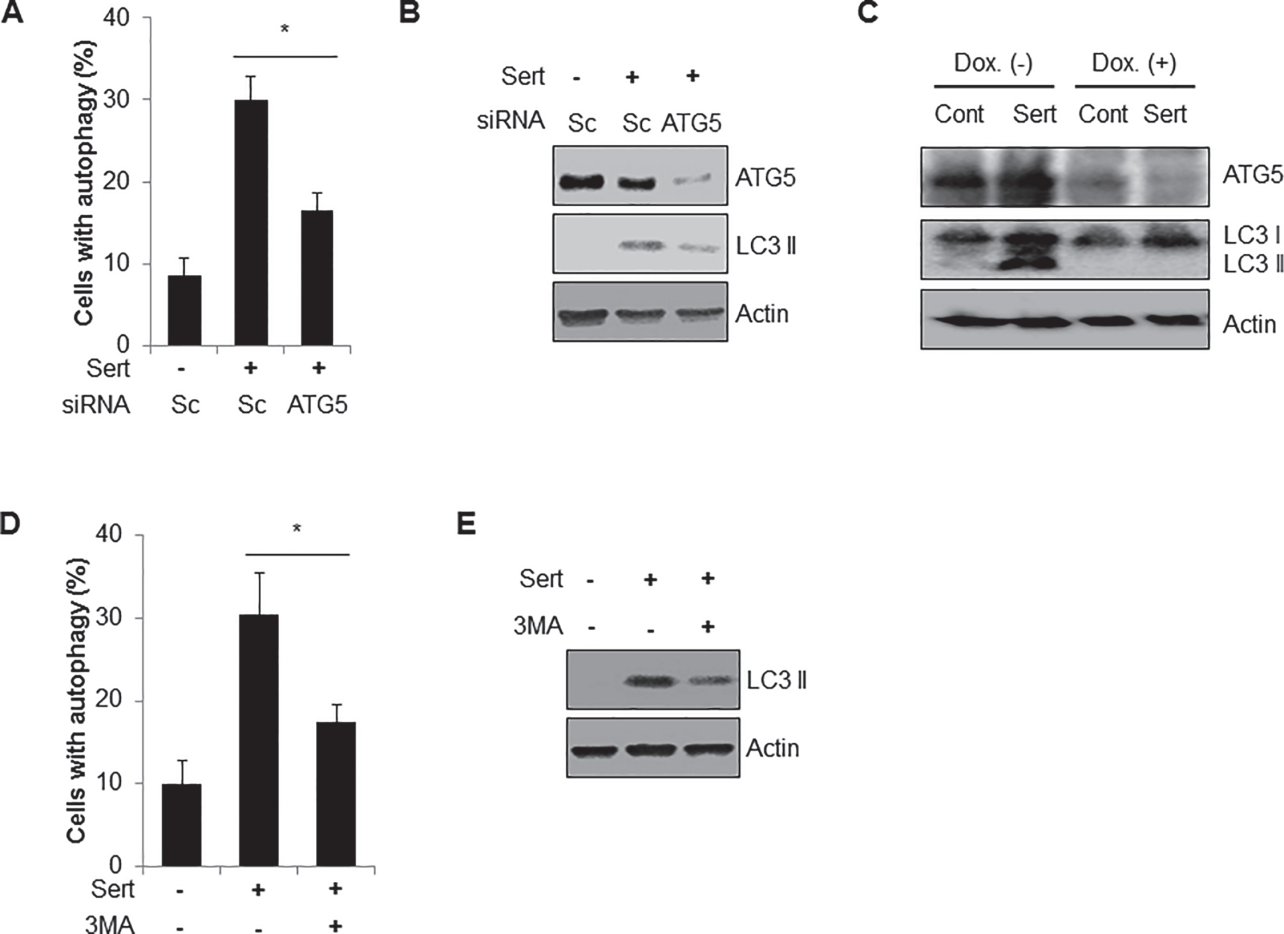
ATG5 mediates sertraline-induced autophagy in htRPE cells. (A and B) htRPE/GFP-LC3 cells were transfected with scrambled siRNA (Sc) or a specific siRNA to *ATG5*. After 3 days, the cells were further treated with sertraline (10 μM) for 24 h. Cells with autophagy were determined by counting the punctate GFP-LC3 dots under a florescence microscope (A). The protein expression levels of LC3 and ATG5 were examined by Western blot analysis (B). (C) The m5-7 MEF cells were treated with Sertraline (10 μM) in the presence or absence of doxycycline (Dox, 10 ng/mL) for 24 h. Then, expression of ATG5 and LC3 was analyzed by Western blotting. (D and E) htRPE/GFP-LC3 cells pre-treated with 3MA (5 mM) for 12 h were treated with sertraline (10 μM) for 24 hr. And cells with autophagy and LC3 protein were examined. Data represent ± standard error of the mean (S.E.M.) from more than three independent experiments (n > 3, * *p* < 0.05).

During the autophagy process, the first step is the formation of an autophagosome. The gene *ATG5* is essential for autophagosome formation. To examine the role of ATG5 in sertraline-mediated autophagy, expression of ATG5 was depleted by RNA interference. Down-regulation of ATG5 significantly decreased sertraline-induced autophagy and LC3|| production (Fig. 4A and B). The effect of ATG5 on sertraline-mediated autophagy was further examined in mouse embryonic fibroblast (MEF) cells (m5-7) which are regulated by the Tet-off system. In the presence of doxycycline (Dox), ATG5 expression was completely suppressed, and autophagy was also inhibited in m5-7 cells [19]. In accordance with our previous results in htRPE cells, treatment of sertraline efficiently induced LC3|| conversion in m5-7 MEF cells (Fig. 4C). However, sertraline failed to induce autophagy in m5-7 MEF cells when Dox was added (Fig. 4C), indicating that ATG5 regulates the sertraline-mediated autophagy. In addition to genetic inhibition, we investigated sertraline-mediated autophagy with a chemical inhibitor, 3MA on sertraline-mediated autophagy. As shown in Fig. 4D and 4E, treatment with 3MA also suppressed the sertraline-mediated autophagy in htRPE cells (Fig 4D and 4E).

### Sertraline-induced autophagy regulates primary cilia formation in htRPE cells

**Fig 5:**
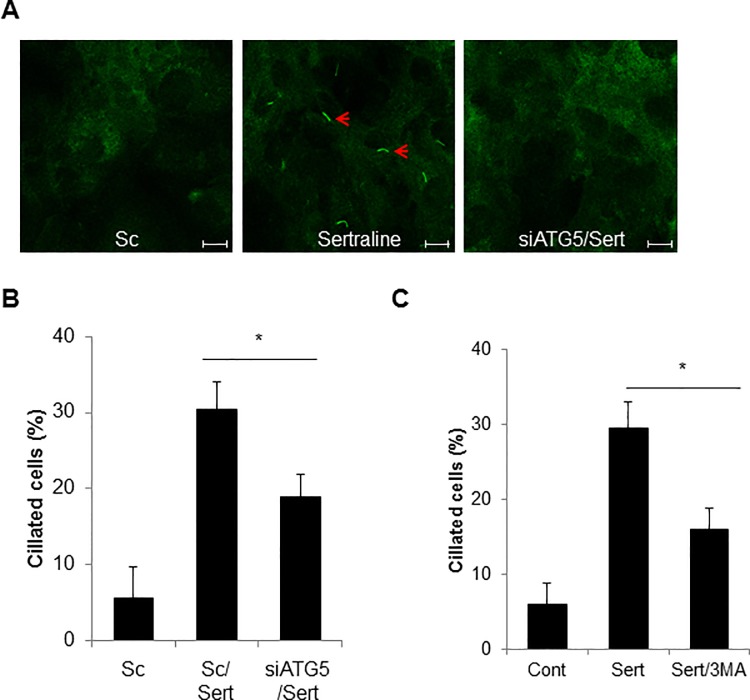
Inhibition of autophagy suppresses sertraline-mediated primary cilia formation htRPE cells. (A and B) htRPE/Smo-GFP cells, which were transfected with scrambled siRNA (Sc) or siRNA against ATG5 (siATG5) were further treated with sertraline (10 μM) for 24 hr. The fluorescence images (A) and ciliated cells were counted under a fluorescence microscopy (B) (Scale bars: 10 μm). (C) htRPE/Smo-GFP cells pre-treated with 3MA (5 mM) for 12 h were further treated with sertraline (10 μM) for 24 h. The ciliated cells were counted under a fluorescence microscope. Data were obtained from at least three independent experiments and values are presented as the means ± S.E.M. (* *p* < 0.05).

Recently, two controversial studies have reported relationship between autophagy and ciliogenesis [25, 27]. Since sertraline induces autophagy as well as cilium formation, we elucidated the effect of sertraline-mediated autophagy on ciliogenesis in htRPE cells. Interestingly, we found that depletion of ATG5 expression significantly reduced the sertraline-induced formation of primary cilium in htRPE cells (Fig. 5A and 5B). Moreover, treatment of an autophagy inhibitor (3-MA) also suppressed the sertraline-induced formation of primary cilium (Fig. 5C). Taken together, our results suggested that autophagy induced by sertraline was involved in ciliogenesis in htRPE cells.

## Discussion

In this study, we showed that sertraline induced both primary cilium formation and autophagy in htRPE cells. Sertraline is one of the most prescribed antidepressants for major depressive disorder in adult outpatients, as well as for obsessive-compulsive, and panic disorders [22]. Sertraline is a SSRI with a high binding affinity to the serotonin transporter, and is more associated with a higher rate of psychiatric side effects such as diarrhea when compared with other selective serotonin reuptake inhibitors [28,29]. Recent evidences suggested that sertraline also possesses other functions which may not be linked with its inhibitory effect on serotonin reuptake. Sertraline down-regulates ATK and has a cytotoxic effect on cancer cells [30,31]. In addition, sertraline blocks persistent late Na^+^ currents in GH3 cells, and suppresses astroglia Kir4.1 channels in HEK293T cells [32,33]. Sertraline also inhibits p-glycoprotein but activates CYP3A [34,35]. Moreover, sertraline enhances cellular calcium levels in various types of cell [36,37]. Increased intracellular calcium is a key signal for many physiological and pathophysiological conditions. The primary cilium is a sensory organelle, which coordinated by cellular signaling such as SHH and Wnt pathway. Two recent reports by the Clapham group have shown that primary cilia are calcium regulated organelles [38,39]. The primary cilium has a high density expression of calcium permeable channels on the membrane, which modulate intraciliary calcium signaling such as SHH [38,39]. However, the effect of sertraline on ciliogenesis was not addressed in these findings. In the present study, we showed that treatment of sertraline strongly induces the formation of cilia but inhibits the disassembly of cilia in htRPE cells (Figs. 1 and 2). We propose that additional investigation the role of calcium in sertraline-mediated ciliogenesis will help to elucidate further the underlying regulation mechanism, since sertraline increases cytosolic calcium levels, which can trigger cilium formation.

Furthermore, studies connecting autophagy and cilliogenesis have been reported recently [25,27,40]. Autophagy is a cellular catabolic event that degrades or recycles cellular components, including cellular organelles. Under normal conditions, autophagy occurs at a basal level, but it can be stimulated in response to various stress conditions such as nutrient starvation, endoplasmic reticulum and oxidative stress, and a lot of pharmacological agents [41]. Tang Z et al. showed that autophagy positively regulates cilliogenesis by autophagic degradation of a ciliopathic protein oral-facial-digital syndrome-1 (OFD1), which is a centriolar satellite protein [22]. In contrast, Pampliega et al. suggested that inhibition of autophagy enhances the growth of primary cilia and cilia-associated signaling while activation of autophagy reduces the growth of cilia [25]. In addition, Choi group showed that autophagy mediates cigarette smoke-induced shortening of cilia and impaired mucociliary clearance (ciliophagy) in respiratory epithelial cells [42]. In this study, we addressed the relationship between autophagy and ciliogenesis. Treatment of sertraline greatly induced autophagy and primary cilium formation in htRPE cells. However, unlike sertraline, Cyto D only activated cilium assembly, but did not induce autophagy (Fig. 3C). Moreover, as shown in Fig. 5, inhibition of autophagy significantly reduced primary cilium formation in cells treated with sertraline. Similar to primary cilium formation, autophagy is also influenced by cytosolic calcium. Calcium is likely to function differently in autophagy. Several groups have shown the inhibitory actions of calcium on autophagy, while others indicated mechanisms of calcium mediating autophagy [43,44]. Thus, we propose that further investigations are necessary to understand the role of calcium in autophagy in sertraline-treated cells.

## Conclusion

In conclusion, although further studies are required to further elucidate the molecular mechanisms underlying the regulation of ciliogenesis, we have shown that autophagy positively modulates ciliogenesis in sertraline-treated htRPE cells.
